# Omega-5-Gliadin Allergy and Cofactors Leading to Anaphylaxis: A Case Report

**DOI:** 10.7759/cureus.81529

**Published:** 2025-03-31

**Authors:** Beatrice Le Bon Chami, Fida Charif, Silvana el Zoghbi, Samir Challita, Fares Zaitoun

**Affiliations:** 1 Pulmonology and Critical Care, Notre Dame Maritime Hospital, Jbeil, LBN; 2 Vascular and Therapeutic Medicine, Centre Hospitalier Universitaire de Saint Etienne, Saint-Etienne, FRA; 3 Pediatric Pulmonology and Allergy, Notre Dame Maritime Hospital, Jbeil, LBN; 4 Pulmonary and Critical Care, Notre Dame Maritime Hospital, Jbeil, LBN; 5 Allergy and Immunology, Rizk Hospital, Beirut, LBN

**Keywords:** allergy and anaphylaxis, cofactor-enhanced food allergy (cefa), food-dependent exercise-induced anaphylaxis (fdeia), omega-5 gliadin (ω-5 gliadin), wheat-dependent exercise-induced anaphylaxis (wdeia)

## Abstract

Anaphylaxis, a potentially life-threatening systemic reaction, is an increasingly common reason for emergency department (ED) admissions, which often poses diagnostic challenges. We present a case of a 62-year-old man who had recurrent anaphylaxis, initially suspected to be triggered by non-steroidal anti-inflammatory drugs (NSAIDs). By conducting thorough history-taking and comprehensive allergy testing, we identified allergic sensitization to the omega-5-gliadin (O5G) component of gluten, which manifested only when wheat consumption was accompanied by specific factors, notably exercise or NSAID use. He was diagnosed eight years after the first anaphylactic episode with wheat-dependent exercise-induced anaphylaxis (WDEIA). Adherence to a gluten-free diet effectively prevented further episodes.

This case underscores the importance of emergency physicians not only in treating anaphylaxis but also in recognizing rare diagnoses, such as cofactor-enhanced food-induced allergic reactions, and understanding their management. This awareness is critical not only for conducting comprehensive assessments in collaboration with allergy specialists but also for facilitating earlier diagnosis, educating patients about the use of epinephrine auto-injectors, and emphasizing essential avoidance measures to prevent the recurrence of anaphylaxis.

## Introduction

Anaphylaxis is a life-threatening hypersensitivity reaction involving multiple organ systems, often presenting with severe cardiovascular and respiratory symptoms. In children, food is a common trigger, whereas in adults, drugs and venom are more frequent [1]. Identifying a single trigger can be challenging, particularly when multiple factors are involved.

Cofactor-enhanced food allergy (CEFA) is a condition in which an allergic reaction occurs only in the presence of additional factors, such as non-steroidal anti-inflammatory drugs (NSAIDs), physical exertion, or alcohol consumption. A rare but well-documented condition is wheat-dependent exercise-induced anaphylaxis (WDEIA), where symptoms appear 30-120 minutes after wheat intake with cofactors [2,3]. Diagnosis can be complex, as patients may tolerate wheat in the absence of cofactors, leading to delays in recognition and treatment [1].

A comprehensive allergy workup, including skin prick tests (SPTs) and specific IgE (sIgE) testing, is crucial for accurate diagnosis and risk assessment. While provocation testing remains the gold standard, its potential risks, such as severe allergic reactions, emphasize the need for careful clinical evaluation before proceeding.

Effective management includes strict avoidance of known triggers, patient education, and emergency preparedness, including the use of epinephrine auto-injectors. In some cases, adopting a gluten-free diet may be the most reliable preventive strategy, as it eliminates exposure to gluten, the primary allergen in WDEIA, reducing the risk of reactions even with cofactors like exercise or NSAIDs [2-4].

This article highlights the importance of comprehensive allergy evaluations in anaphylaxis cases, emphasizing the need to identify allergens and cofactors to prevent potentially fatal reactions.

## Case presentation

A 62-year-old man presented to our emergency department (ED) on three separate occasions due to recurrent anaphylaxis of unknown origin. His medical history included seasonal rhinitis, recurrent urticaria and pruritic reaction, regular use of NSAIDs for back pain, occasional use of proton pump inhibitors (PPIs), and alcohol consumption. He had never undergone allergy testing.

On the first visit to the ED, he presented with hypotension (60/40 mmHg), generalized hives, flushing, and pruritus approximately one hour after dinner. The symptoms were resolved after the administration of intramuscular (IM) epinephrine. He was monitored in the hospital and discharged the following day. No underlying cause was identified at that time.

Six years later, he experienced an episode of anaphylaxis after consuming various foods, including wheat. Earlier that day, he had taken 500 mg of naproxen twice in the morning and a PPI. Approximately one hour after lunch, he developed generalized hives and pruritus. On arrival at the ED, he was dyspneic, agitated, and sweating profusely, with unmeasurable blood pressure. His oxygen saturation on room air was 96%. He was treated with three doses of epinephrine, a normal saline bolus, and steroids and was subsequently admitted to the intensive care unit. He was discharged on the second day. Despite the patient’s history of taking NSAIDs without prior allergic reactions, which should have guided physicians toward a different diagnosis, he was diagnosed with an NSAID allergy and advised to avoid them.

Two years after this episode, and 30 minutes after a pasta meal, the patient went for a vigorous walk, during which he experienced generalized urticaria, facial edema, and pruritus but no hypotension. In the ED, he was treated with intravenous (IV) promethazine, with good clinical response, and was discharged home.

During a thorough assessment, SPTs showed positive reactions to wall pellitory pollen and house dust mite. Wheat was not included in the testing. As presented in Table 1, sIgE testing with a component-resolved molecular diagnostic assay revealed significantly elevated titers to the Par j allergen component of wall pellitory pollen, explaining the patient’s allergic rhinitis. Additionally, high titers to the Tri a 19 (omega-5-gliadin) (O5G) component of wheat indicate a wheat allergy. No correlation was observed with allergens present at low IgE levels.

**Table 1:** Allergy Xplorer 1 results > 0.3 kUA/L nsLTP: non-specific lipid transfer protein Par j 1 and Par j 2: specific allergens from *Parietaria judaica* (wall pellitory) Sec_flour: *Secale *flour (cultivated rye) Tri a 19: *Triticum aestivum* (common wheat) Equ c_milk: mare’s milk (*Equus caballus*) Hom g: *Homarus gammarus* (European lobster) Loc m: *Locusta migratoria* kUA/L: kilo units of allergen per liter <0,3 kUA/L: 0, negative or uncertain 0,3-1 kUA/L: 1, low IgE Level 1-5 kUA/L: 2, moderate IgE Level 5-15 kUA/L: 3, high IgE Level >15 kUA/L: 4, very high IgE level

Detectable sensitizations				
Category	Name	Allergen	Function	kUA/L
Pollens	Wall pellitory	Par j	-	2.51
		Par j 2	nsLTP	10.59
Food	Cultivated rye	Sec_flour	-	0.85
	Common wheat	Tri a 19	O5G	9.27
	Mare’s milk (horse milk)	Equ c_milk	-	0.4
	Lobster	Hom g	-	0.81
	Migratory locust (meat)	Loc m	-	0.31

Based on a comprehensive review of the patient’s history and elevated IgE levels to O5G, a diagnosis of WDEIA was made. However, no food challenge was conducted.

Despite potentially tolerating wheat in the absence of cofactors such as NSAIDs or exercise, the patient decided to completely eliminate wheat from his diet. He was advised to carry an epinephrine auto-injector at all times for potential future anaphylactic reactions.

Since eliminating gluten from his diet, the patient has had no major allergy relapses. However, five years later, he experienced an allergic reaction with urticaria and pruritus 90 minutes after consuming whisky and a hamburger during a 6 km walk. His symptoms resolved within hours after self-administering oral antihistamines and corticosteroids.

## Discussion

Allergic food reactions in adults, especially those of new onset or previously undiagnosed, carry a high clinical risk as they often present with anaphylaxis of varying severity.

Fatal and near-fatal cases are uncommon and unpredictable, highlighting the importance of proficiency in recognizing, diagnosing, and effectively managing anaphylaxis [5]. However, identifying the source of food-induced anaphylaxis (FIA) can be challenging, especially when cofactors are involved, which occurs in more than half of the cases [6].

Exercise is the most common cofactor for FIA. “Food-dependent exercise-induced anaphylaxis” (FDEIA) is used when anaphylaxis occurs during or shortly after exercise and is dependent on the ingestion of an offending food [7]. Moderate or even light exercise, such as walking or gardening, is a sufficient cofactor. Other cofactors are alcohol and NSAIDs, followed by acetylsalicylic acid (ASA), PPIs, acute infections, stress, fatigue, heat, pollen peaks, perimenstrual status, and sleep deprivation [8,9]. Cofactors contribute to FIA by decreasing the threshold of allergen exposure needed to trigger anaphylaxis and by amplifying the risk of anaphylaxis in patients with low or borderline allergen sensitization [3,8,10,11]. The underlying immunological mechanisms are complex and not fully understood, involving multiple pathways and potentially interconnected pathogenic processes [5,7,12-14]. Exercise, alcohol, and NSAIDs are believed to enhance allergen absorption by increasing intestinal permeability and disrupting the gut barrier, resulting in a greater exposure of mast cells to allergens. However, evidence indicates that exercise-induced changes in gut permeability occur primarily only after prolonged or high-intensity physical activity [15]. Suppression of gastric acids by PPIs may lead to food allergens’ persistence [10]. Additionally, cofactors may activate mast cells directly or through the release of mediators, leading to an exaggerated immune response in sensitized individuals [3]. Exercise is thought to enhance mast cell activation by increasing plasma osmolarity, redistributing blood flow, and activating adenosine and eicosanoid metabolism [15]. The cofactor effect of NSAIDs has been related to cyclooxygenase inhibition and, therefore, prostaglandin E2 production. Alcohol has been associated with an increase in histamine levels by inhibition of diamino oxidase and also with an increase in extracellular adenosine by inhibition of its uptake [6,15].

In CEFA, 99% of triggers are plant-derived, mainly cereals and vegetables. O5G protein found in wheat, also called Tri a 19, is the most common trigger in adults and tends to cause more severe reactions compared to other food allergens [2,4,11]. When wheat is identified as the causative agent, the condition is termed “wheat-dependent exercise-induced anaphylaxis” (WDEIA), a rare but well-documented disorder.

In WDEIA, the symptoms and the severity of allergic reactions can vary significantly across episodes in the same patient. Wheat is typically tolerated in most instances without triggering a reaction, yet reactions occur intermittently and inconsistently following wheat ingestion, often without an obvious cause. This pattern appears to be a distinguishing characteristic of O5G allergy in adults, setting it apart from other forms of classical type 1 food allergy. It remains unclear whether the variability in reactions is influenced by the quantity of O5G ingested [16].

Symptoms typically appear 30-120 minutes after eating and 10-50 minutes into exercise, or if food is consumed shortly after exercise [2,3]. Compared to classical food allergy, the time-to-onset of reactions is longer in WDEIA [2-4,13,17]. Nearly half of wheat-induced reactions involve cofactors other than exercise, such as NSAIDs or alcohol [1,13,17,18]. Exercise, alcohol, and multiple cofactors were more frequent in males. Exercise, pollen peak, and multiple cofactors are more often related to anaphylaxis [3,11]. Symptoms can range from pruritus and urticaria, which are almost invariably the earliest signs, to anaphylaxis. Most patients have experienced multisystem anaphylaxis at least once [4,16]. Notably, anaphylactic reactions in FDEIA tend to be more severe than those seen in typical IgE-mediated FIA because anaphylaxis more often manifests with cardiovascular symptoms, such as hypotension and loss of consciousness, while respiratory symptoms are less commonly reported [2,6,11,19]. A history of prior anaphylaxis is a significant risk factor for future anaphylaxis [12].

O5G allergy also differs from other food allergies in that it is largely adult-onset, peaking in the third to fifth decades of life. Studies have reported either a balanced sex ratio or a slight predominance of males [2,13,16,17]. O5G allergy is also associated with lower rates of atopic comorbidities, such as asthma, allergic rhinitis, or atopic dermatitis, than other food allergies. Some patients may have coexisting allergies to other foods, such as shellfish or nuts [2-4,13]. The patient had high sIgE to *Parietaria*, which explains his allergic rhinitis [17]. However, although there is no known cross-reactivity between wheat and *Parietaria* pollen [11], it has been described that patients with a history of atopy, particularly allergic rhinitis, may be at higher risk of recurrent FDEIA [19]. The patient also had a low level of sIgE to lobster but never had an allergic reaction after eating shellfish. He showed a low level of sIgE to cultivated rye. Although a possible cross-reactivity between O5G and cultivated rye (secalins) has been identified, clinical reactions to rye have not been observed in WDEIA patients [16].

Delayed diagnosis of WDEIA is common, with some patients remaining undiagnosed for over five years, underscoring the need for improved screening and identification by clinicians. Initially, other conditions are often misdiagnosed, including exercise-induced urticaria/anaphylaxis, idiopathic anaphylaxis, and recurrent acute urticaria [16].

Patients with suspected O5G allergy should be referred to an allergy specialist for confirmation of diagnosis and management. Diagnostic tools include SPTs, sIgE testing analysis of multiple molecular components, and provocation tests [3,14,16]. SPTs are usually used as a first-line test to diagnose IgE-mediated allergies as they are quick, less invasive, and cost-effective compared to blood tests. SPTs are preferably performed with gluten or gliadin, as SPT responses are known to be more specific and sensitive than those with wheat flour [12,14]. sIgE tests identify sensitization to specific allergens but not always clinical symptoms. Molecular IgE is an advanced form of sIgE testing that identifies IgE against specific protein components of an allergen (e.g., Par j in wall pellitory allergy or Tri a 19 in wheat allergy). Microarrays like ImmunoCAP and ALEX2 analyze over 120 allergen extracts and 170 molecular components, significantly enhancing IgE repertoire analysis and providing valuable insights for complex cases. Testing for sIgE to O5G should be considered in patients presenting with exercise-induced urticaria/anaphylaxis, idiopathic anaphylaxis, and recurrent acute urticaria. Notably, the level of sIgE to O5G does not predict the severity or frequency of allergic reactions [16,17]. Provocation tests confirm or rule out allergies by exposing patients to suspected allergens and are essential when other tests are inconclusive or when clinical history and test results differ. A positive test triggers objective symptoms, confirming allergic reactivity. The placebo-controlled wheat/exercise challenge is a key diagnostic tool, though currently lacking standardization in factors like wheat/gluten dose, exercise intensity, and cofactor use such as ASA and ethanol [2,3,12,14,18]. They pose significant risks, including anaphylaxis, making them impractical in some cases. Due to sIgE for O5Gs near 100% specificity, some consider its presence sufficient for WDEIA diagnosis [16].

In our case report, neither the patient nor the physicians initially linked wheat consumption to his allergic symptoms, which were more severe when cofactors were involved, particularly NSAIDs and exercise. The patient’s regular use of NSAIDs and PPIs, together with a history of recurrent pruritus and urticaria, may have helped to diagnose WDEIA earlier. Ultimately, the analysis of multiple molecular components led to the correct diagnosis. In agreement with the patient, we decided not to perform a provocation test, given the patient’s history of anaphylactic shock, as the diagnosis was already clear. For WDEIA, stopping physical activity at the first symptoms is crucial to prevent worsening [3]. Patient education is key, though avoiding cofactors like exercise, NSAIDs, or infections is challenging, as infections are particularly challenging since they are frequent in daily life [6,12]. In mild cases, reducing wheat intake before exercise and avoiding other triggers may suffice [3]. A gluten-free diet remains the most effective prevention [2,4], yet unexpected exertion and accidental gluten intake, especially when dining out [4], can still cause reactions, as seen in our patient five years post-diagnosis. Therefore, prescribing antihistamines, steroids, and an epinephrine autoinjector is essential [12,16,17]. Ongoing trials on wheat oral immunotherapy aim to improve safety and efficacy [20], while reducing O5G content in wheat products may offer future alternatives [9].

## Conclusions

WDEIA is a rare but serious food allergy that is often delayed in diagnosis or misidentified as conditions like exercise-induced or idiopathic anaphylaxis. The presence of cofactors, particularly exercise and NSAIDs, significantly influences symptom severity, ranging from mild urticaria to life-threatening anaphylaxis. Recognizing both the triggering allergen and its cofactor dependency is crucial for accurate diagnosis, risk assessment, and the prevention of severe reactions.

The primary allergen responsible for WDEIA, O5G in wheat gluten, can be detected through skin prick and molecular IgE testing, which highly supports a WDEIA diagnosis. However, provocation tests, while considered the gold standard, should be used cautiously and only in cases of diagnostic uncertainty due to their inherent risks.

As seen in most cases, the patient experienced significant symptom improvement after adopting a gluten-free diet, reinforcing the essential role of dietary management. However, since anaphylaxis can still occur despite dietary precautions, carrying an epinephrine autoinjector remains essential.
